# IRVING S. WRIGHT AWARD AND TERRIE FOX WETLE AWARD LECTURES

**DOI:** 10.1093/geroni/igae098.1116

**Published:** 2024-12-31

**Authors:** Hattie Herman

**Affiliations:** Chair

## Abstract

The Irving S. Wright Award of Distinction Lecture will feature an address by the 2024 recipient Jeremy D. Walston, MD, FGSA, Johns Hopkins University. The Terrie Fox Wetle Award lecture will feature an address by the 2024 recipient Megan Huisingh-Scheetz, MD, MPH, AGSF, University of Chicago. The George M. Martin Lifetime Achievement in Mentoring Award will also be presented at this event to 2024 recipient Steven N. Austad, PhD, FGSA, University of Alabama at Birmingham. These awards are given by the American Federation for Aging Research, Inc.

